# Proteins and Enzymes from Marine Resources

**DOI:** 10.4061/2011/594646

**Published:** 2011-11-20

**Authors:** Nabil Miled, Moncef Nasri, Hideki Kishimura, Faouzi Ben Rebah

**Affiliations:** ^1^LBGEL, ENIS, Route Soukra, Sfax 3038, Tunisia; ^2^Biotechnology Department, ISBS, Route Soukra, Sfax 3038, Tunisia; ^3^LGEM, ENIS, Route Soukra, Sfax 3038, Tunisia; ^4^Faculty of Fisheries, Hokkaido University, Hakodate 041-8611, Japan

Sea products are valuable resources of natural substances such as lipids, polysaccharides, enzymes, vitamins, and proteins. In this issue, a variety of fish proteases (trypsin, alcalase, etc.) have been characterized and sometimes purified. Many of these enzymes display potentially interesting new biochemical properties for industrial applications. Their potential adequacy for biotechnological applications such as chitin extraction was demonstrated. Biochemical characteristics of crude alkaline protease extracts from the viscera of goby (*Zosterisessor ophiocephalus*), thornback ray (*Raja clavata*), and scorpionfish (*Scorpaena scrofa*) were studied. At least four caseinolytic proteases bands were observed in zymogram of each enzyme preparation. These proteolytic preparations were successfully used in the deproteinization of shrimp wastes for chitin extraction. Furthermore, a trypsin was purified from the pyloric ceca of Pacific cod (*Gadus macrocephalus*) using chromatographic methods. The cod trypsin was successfully applied to catalyze dipeptide synthesis using series of “inverse substrates.” A Mackerel trypsin was also purified from defatted viscera by supercritical carbon dioxide, and its biochemical properties were studied.

Recent biotechnological progresses require the research of new bioactive peptides from natural resources that can be used as an alternative to chemical products in various applications (food, cosmetics, drugs, etc.). Sea products proteins digestion using bacterial proteases generated protein hydrolysates displaying interesting biochemical properties and antioxidant activity. Antioxidative activities and biochemical properties of protein hydrolysates with different hydrolysis degrees, prepared from cuttlefish (Sepia officinalis) using an Alcalase and *Bacillus licheniformis* NH1 proteases were determined. The antioxidant activities of cuttlefish protein hydrolysates increased with increasing the hydrolysis degree. Antioxidative activity was concentration dependent. Furthermore, both Alcalase and NH1 protein hydrolysates were able to retard lipid peroxidation and *β*-carotene-linoleic acid oxidation. In addition, cuttlefish protein hydrolysates have a high percentage of essential amino acids and could be used as supplement to poorly balanced dietary proteins. Furthermore, carbohydrate-binding lectins were also extracted from many marine resources.

This issue highlights the potential of sea products as valuable resources of bioactive peptides and proteins such as hydrolytic enzymes, lectins, and antioxidants. These resources might be of great interest for industrial biotechnological processes using safe and natural materials such as peptides or enzymes. 

Dicer is an RNase III enzyme with two catalytic subunits, which catalyzes the cleavage of double-stranded RNA to small interfering RNAs and micro-RNAs, which are mainly involved in invasive nucleic acid defense and endogenous genes regulation. In addition, Dicer is thought to be involved in defense mechanism against foreign nucleic acids such as viruses. A paper focused on the recent progress of Dicer-related research and discussed potential RNA interference pathways in aquatic species. Dicer is abundantly expressed in embryos, indicating the importance of the protein in early embryonic development. 

*Nabil Miled* *Nabil Miled* *Moncef Nasri* *Moncef Nasri* *Hideki Kishimura* *Hideki Kishimura* *Faouzi Ben Rebah* *Faouzi Ben Rebah*

